# *Preventing Chronic Disease*: Progress and Achievement of 2017 Priorities

**DOI:** 10.5888/pcd14.170425

**Published:** 2017-10-12

**Authors:** Leonard Jack

**Figure:**
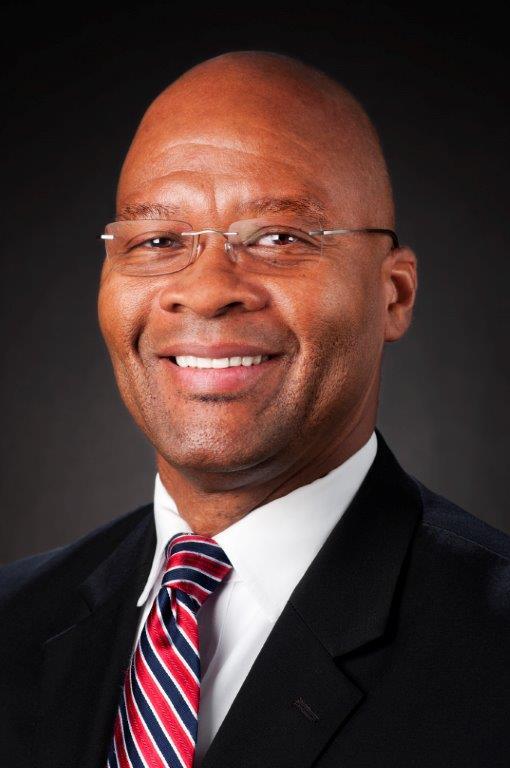
Leonard Jack, Jr, PhD, MSc, Editor in Chief

It was a remarkable year for *Preventing Chronic Disease* (*PCD*) as we embarked on a journey to advance the journal’s mission and vision and achieve priorities. I was appointed editor in chief of *PCD* in October 2016, and this column marks my one-year anniversary. My first column, in February, established that I would publish 3 columns this year to announce actions and appointments, discuss timely public health topics, and update readers on the journal’s priorities. I also identified 10 priorities for the journal in 2017:

- Adopting a multilayered approach to peer review
- Securing additional associate editors
- Promoting global public health perspectives
- Ensuring scientific integrity
- Publishing collections of articles
- Accelerating the dissemination of critical research
- Introducing a new article type to focus on implementation evaluation
- Maintaining the Student Research Paper Contest
- Developing tools and resources for novice authors
- Enhancing our brand as an innovator in scholarly publishing

In this final Editor in Chief’s Column for 2017, it is important to me to provide *PCD* readers with updates on each of these priorities, toward which considerable progress has been made.

## Adopting a Multilayered Approach to Peer Review

*PCD* carefully examined its peer-review process, and thanks to an expanded team of associate editors, we added an additional quality control stage to the process. Each manuscript is first screened to ensure it aligns with the journal’s mission and publication standards. A manuscript that meets these initial submission requirements is assigned by me to one of our associate editors, who determines whether the manuscript should undergo peer review. The associate editor then makes a recommendation on the manuscript on the basis of his or her assessment of the peer reviews, and as editor in chief I make the final decision. In addition to adding a new layer to our peer-review process, we began using a rating system to evaluate the quality of reviews, so associate editors can select the best reviewers and ensure that manuscripts are assessed by highly qualified, independent, and experienced peer reviewers. 

This multilayered peer-review process established important quality control checkpoints, positioned authors to receive valuable feedback on the scientific and programmatic content of their manuscripts, and improved the quality of manuscripts accepted for publication. In addition, these enhancements influenced the journal’s acceptance rate, which decreased from 32.5% from January through August 2016 to 26.9% for the same period in 2017.

## Securing Additional Associate Editors

*PCD* assembled an impressive group of researchers and practitioners capable of recommending manuscripts for publication on the basis of merit, appropriateness, and the highest ethical standards in scholarly publishing. In October 2016, *PCD* had 3 associate editors. Since then, *PCD* has appointed 16 new associate editors with experience in population health, health systems, implementation evaluation, geographic information systems, health economics, behavioral health, and applied epidemiology. To ensure the stability of the journal’s institutional knowledge while still allowing room to recruit new expertise to meet future research and programmatic priorities, we established appointment terms of 2 years, 3 years, and 4 years. All associate editors undergo training on the journal’s mission, direction, and publication standards for all article types; on the logistics of the journal’s manuscript system; and on how to provide meaningful feedback to authors. The increase in the number of associate editors in critical research areas has assisted the journal in providing relevant and useful feedback to authors, reducing manuscript review time, and improving the quality of manuscripts submitted and accepted for publication. To learn more about the background and experience of our associate editors, please see www.cdc.gov/pcd/about_the_journal/associate_editors.htm.

## Promoting Global Public Health Perspectives

*PCD* recognizes that understanding the conditions that shape health in various settings and geographical locations is critical to advancing public health and medicine. *PCD* established a priority to serve as a resource to researchers and practitioners internationally, recognizing that factors influencing health in the United States are similar to those in other countries. We have worked closely with our editorial board to identify strategies to increase submissions from experts who are conducting population health interventions that address diabetes, cancer, cardiovascular health, and other public health topics in other countries. From January through August 2017, the journal received 89 submissions from countries outside of the United States — including Australia, Canada, Germany, Guatemala, Israel, Mexico, Peru, Portugal, and Taiwan — an increase of 20 manuscripts from this same period in 2016. Ten of these manuscripts, mostly original research articles, were accepted for publication. Readers can anticipate articles describing international efforts to prevent and control diabetes, education of children about stroke prevention, use of geographic information systems technology to monitor multiple chronic conditions, and how public health surveillance systems are being used to monitor and track health conditions.

## Ensuring Scientific Integrity

*PCD* recognizes that several factors can contribute to situations of scientific misconduct — lack of confidence, pressure to publish in established journals, competition within disciplines and professions, desire for prestige and recognition, lack of consequences established by the journal, and poor research and writing skills. *PCD* takes the matter of ensuring scientific integrity seriously, and we took steps to provide our editorial board, associate editors, peer reviewers, and authors with guidance on the journal’s expectations for preparing and disseminating findings to the public health community. We have sought to educate them about the need to ensure scientific integrity at all levels: analyzing and reporting results, determining authorship, acknowledging and obtaining approval to use copyrighted material, recognizing how previous research helped to advance reported findings, and disclosing conflicts of interests. Information on *PCD*’s policy on scientific integrity was refined and can be reviewed on our Editorial Policy page: www.cdc.gov/pcd/about_the_journal/editorial_policy.htm.

## Publishing Collections of Articles

*PCD* is often approached by authors and organizations interested in publishing multiple articles that address a common theme. In November 2016, we released new guidance on submitting proposals for collections. Articles published in *PCD* collections undergo the same rigorous peer-review process as regular submissions to ensure that their scientific and programmatic content meets the highest standards of scholarly research. During the past year, we worked closely with authors of articles in 2 collections.

The first collection, “State and Local Public Health Actions to Prevent and Control Chronic Disease,” recognizes that chronic diseases often occur simultaneously and result from interrelated risk factors. This collection showcases how 4 programs at the Centers for Disease Control and Prevention (CDC) — diabetes; heart disease and stroke prevention; nutrition, physical activity, and obesity; and school health — can work together to address state and local efforts to improve health outcomes. Articles in this collection describe the use of 3 primary strategies: 1) improving environments and settings to promote positive health behaviors; 2) improving the delivery and use of quality clinical services and other health services to prevent and manage high blood pressure and diabetes; and 3) increasing links between communities and clinical organizations to support prevention of diabetes, high blood pressure, and obesity.

Childhood obesity is a global public health problem. Obese children are at high risk for becoming obese adolescents, placing them at risk for developing chronic diseases later in life. In addition, obese children are more prone to have emotional and psychological challenges than are children who are not obese. The second collection, “The Childhood Obesity Research Demonstration (CORD) Project: Real-World Implementation of Multisetting Interventions to Address Childhood Obesity,” focuses on real-world implementation of evidence-based interventions in multiple settings — schools, early child care and education centers, health care facilities, and the community — with the goal of improving healthy eating and physical activity among low-income children.

Articles in these 2 collections describe original research not previously published in indexed scientific literature. *PCD* encourages authors and organizations interested in reviewing previously published collections and finding guidance on submitting a proposal to visit the Collections section of our website: www.cdc.gov/pcd/collections/index.htm.

## Accelerating the Dissemination of Critical Research

Throughout this year, we identified several ways to accelerate the dissemination of critical research: streamlining author submission requirements; identifying and securing reviewers with a track record of high-quality and timely peer reviews; and emphasizing promotion of the Research Brief. A Research Brief is essentially a shortened version of an Original Research article. It offers authors an opportunity to have their work reviewed more quickly and published in a shorter turnaround time. From January through August 2017, the journal received 65 Research Brief submissions, and 19 of these manuscripts were accepted for publication. The accepted Research Briefs cover diverse topics, such as the role of age and acculturation in diabetes management, aspirin use for the primary and secondary prevention of cardiovascular disease, prevalence and correlates of sleep apnea among US veterans, and concordance of blood-pressure–control estimates using thresholds derived from treatment guidelines. We will continue to identify ways to publish rigorous research and evaluation findings — by using quality controls, modifying the formats of article types, enlarging the pool of experienced and reliable reviewers, and maintaining a robust management system — to quickly move manuscripts through all stages of review, production, and publication.

## Introducing a New Article Type to Focus on Implementation Evaluation

In March 2017, *PCD* developed a new article type, Implementation Evaluation, which provides readers with information on how public health programs and interventions are developed and evaluated on the basis of diverse factors (ie, program focus, population characteristics, staffing capacity, geographic location, patient–community–health system partnerships, and fiscal resources) in real-world settings. Implementation Evaluation articles offer an opportunity for authors to present results from tailored, setting-specific evaluation methods and approaches. As such, Implementation Evaluation articles are positioned to discuss how evidence-based programs and interventions can be adapted in other settings. Since its launch in March 2017, *PCD* has received 10 submissions that examine effective ways to integrate population-based interventions, practices, and evaluation methods into routine clinical, public health, health care, school, worksite, and community settings.

## Maintaining the Student Research Paper Contest

*PCD* is committed to providing opportunities for young researchers to contribute to public health and develop critical writing and reviewing skills. We are pleased to showcase in this week’s release the winners of the 2017 Student Research Paper Contest. This year, instead of having one winner, we introduced 4 categories of winners: high school, undergraduate, graduate, and doctoral. We have 5 winners this year, rather than 4, because the caliber of submissions in the doctoral student category was so high that our panel decided on 2 winners. Special thanks go to our editorial board for their assistance in serving on the reviewing panels to identify this year’s winners.

It is important to note that student researchers, regardless of category, had to meet our standard to serve as first authors — that is, the person who conducted or led the research described in the manuscript and prepared the first draft. *PCD* received a record 72 contest entries this year, compared with 37 in 2015 and 59 in 2014. We were thrilled to see this number of high-quality entries. We recognize that these entries would not have been possible without the support of faculty mentors working closely with students to develop their research competencies and scientific writing skills. Although we could not name every entry as a winner, we thought several nonwinning submissions were of sufficient merit to warrant peer review. *PCD* has accepted and will publish a total 19 manuscripts, all of which will be available soon on *PCD*’s Collections page. For more information on this year’s contest and access to the 5 winning student research articles, please see this week’s Table of Contents: www.cdc.gov/pcd/index.htm. For information on our 2018 Student Research Paper Contest rules and expectations, please visit our Announcements page: www.cdc.gov/*PCD*/announcements.htm. Student entries must be received electronically no later than 5:00 PM EST on Friday, February 23, 2018.

## Developing Tools and Resources for Novice Authors

*PCD* is committed to assisting authors with tools and resources for strengthening skills and building confidence in scientific writing. This year our editorial staff reviewed and revised the journal’s instructions for authors, and the updated content is provided in our new “Author’s Corner”: www.cdc.gov/*PCD*/for_authors/index.htm. In addition, editorial staff members updated information on *PCD*’s Types of Articles page and provided detailed information for authors on the requirements for each: www.cdc.gov/*PCD*/for_authors/types_of_articles.htm.

*PCD* finalized a new section on the following topics: 1) Top 5 Reasons Manuscripts Are Rejected; 2) Top 5 Mistakes in Tables; 3) Top 5 Mistakes in Reporting Statistics; and 4) Top 5 Mistakes in Figures. This new section was developed on the basis of *PCD*’s years of experience in working with authors to improve manuscripts as well as guidelines published in the *AMA Manual of Style: A Guide for Authors and Editors — 10th Edition* and other resources describing how to present statistical information to medical and public health audiences. The section was designed to help authors to position their manuscripts for acceptance. Authors can access this guidance by visiting the Author’s Corner: www.cdc.gov/pcd/for_authors/index.htm.

## Enhancing Our Brand as an Innovator in Scholarly Publishing

*PCD* is committed to enhancing the journal’s brand as an innovator in scholarly publishing. During the past 4 months, *PCD* implemented a Simplified Submission pilot program to reduce the amount of time authors spend in the process of submitting manuscripts. The pilot program has been a tremendous success; instances of manuscripts being returned to authors for formatting corrections have dropped from 45 to 4 since the program began, and manuscript check-in time has decreased from 1.12 days to 0.68 days.

*PCD* has introduced another pilot program to offer opportunities for *PCD*’s top peer reviewers to receive continuing education (CE) credits. The pilot is only one of the avenues *PCD* is exploring to recognize excellence among our peer reviewers in providing thoughtful, comprehensive, and timely reviews. *PCD* is now looking into recognizing reviewers for their excellence through their ORCID ID or Publons profile — interactive mechanisms that link people with their professional activities. As editor in chief I have made my own commitment this year to recognize these important contributions, and I have emailed many of our top reviewers to express my appreciation for the quality of their reviews and emphasize how critical these reviews are to the journal’s success.

*PCD* has increased efforts to promote articles to the news media by distributing media summaries through the CDC Newsroom (www.cdc.gov/media/PCD/index.html), increasing social media outreach, and releasing podcasts. *PCD* rebranded its podcasts as “*PCD* Sound Bites” and developed and introduced a new logo. To track the success of these outreach efforts, *PCD* is working to identify additional metrics to monitor the uptake, citation, impact, and visibility of *PCD* articles.

## Conducting External Review to Assess Journal

And finally, perhaps *PCD*’s biggest achievement in working to improve its brand is conducting the journal’s first-ever external review. *PCD* invited a distinguished panel of health and medical professionals to assess the journal’s progress and to identify ways to strengthen *PCD*’s ability to disseminate cutting-edge programmatic, research, and evaluation findings. Although *PCD* has always been considered a valuable resource to a broad audience of readers, we are ready to take the journal to the next level. In June 2016, we invited 7 experts in public health, scientific writing, and scholarly publishing to examine the journal’s strengths and areas of growth and answer a series of fundamental questions about the journal’s focus and direction. The panel provided their expert opinions on *PCD*’s mission, scientific quality, scope, audience, editorial standards and protocols, metrics and analytics, distribution platforms to communicate with readers, and future priorities. *PCD* is reviewing feedback from the external review panel’s report, and during the next 3 months we will engage in discussions with our editorial board and associate editors, CDC’s senior leadership, and *PCD* staff members to refine the journal’s mission statement and vision statement. We will redefine the primary topics of interest and decide which topic areas the journal will no longer consider. We will share more details about the journal’s new direction with our readership early next year. Please stay tuned for exciting enhancements geared to uphold *PCD*’s commitment to be an influential journal promoting the best science and practice in public health.

## Final Thoughts and Acknowledgments

This year has been one of tremendous progress and success for the journal. We are committed to securing the necessary expertise among the journal’s editorial board and associate editors to recognize the best scientific and programmatic contributions in public health. *PCD* will continue to identify and implement ways to improve our authors’ interaction with every aspect of the journal, from submission to publication, and provide timely guidance so that authors have the best chance of receiving favorable peer reviews. For the growing number of student authors, the journal created a more level playing field in the Student Research Paper Contest by establishing categories for different levels of education. *PCD* continued its brand as an innovator, from improvements in the journal’s website and submissions process, to creative ways to recognize our top peer reviewers, to developing the best processes to ensure timely, courteous, and responsive service from *PCD* staff members.

*PCD* could not have achieved these 10 priorities without a carefully considered plan of action. Furthermore, *PCD* could not have achieved them without the shared support of readers, authors, peer reviewers, editorial board members, associate editors, staff members, and senior leadership in CDC’s National Center for Chronic Disease Prevention and Health Promotion. Thanks to these many people for their unwavering support of the journal.

